# Ultrasound–Fluoroscopy Versus Ultrasound Guidance for Catheter Drainage of Loculated Pleural Effusions: A Retrospective Study

**DOI:** 10.3390/diagnostics16132089

**Published:** 2026-07-03

**Authors:** Inae Hwang, Jaeyoon Kim, Pa Hong, Yangwon Kim

**Affiliations:** Department of Radiology, Samsung Changwon Hospital, Sungkyunkwan University School of Medicine, Changwon 51353, Republic of Korea

**Keywords:** percutaneous drainage, pleural effusion, ultrasonography, fluoroscopy, reintervention, catheterization, radiology, interventional

## Abstract

**Background:** Loculated pleural effusion—characterized by fibrinous septations that compartmentalize the pleural space and impede drainage—is a technically demanding indication for percutaneous catheter drainage (PCD), in which accurate catheter placement directly determines drainage success. Whether fluoroscopic guidance reduces reintervention compared with ultrasound guidance alone in this population is unknown. **Methods:** We conducted a retrospective cohort study of 190 PCD procedures for loculated pleural effusion at a single tertiary-care center (Samsung Changwon Hospital, Changwon, South Korea; 118 ultrasound-guided [US alone], median age 71 years; 72 combined ultrasound-and-fluoroscopy-guided [US + Fluoroscopy], median age 75 years). Underlying etiologies included parapneumonic effusion, empyema, malignant effusion, and other causes. The primary outcome was any reintervention. Between-group comparisons used the Mann–Whitney U and Fisher’s exact tests; adjusted analyses included multivariable logistic regression, 1:1 propensity score matching (PSM), and Cox proportional hazards modeling. **Results:** Reintervention occurred in 35.6% of US alone versus 18.1% of US + Fluoroscopy procedures (relative risk 0.51, 95% CI 0.29–0.88; *p* = 0.013). After adjustment, US + Fluoroscopy was associated with lower odds of reintervention (adjusted OR 0.42, 95% CI 0.17–0.95; *p* = 0.043). PSM (60 matched pairs) confirmed this finding (35.0% vs. 16.7%; McNemar’s *p* = 0.046). The Cox model showed a directionally consistent association (adjusted HR 0.50, 95% CI 0.25–1.02; *p* = 0.056; log-rank *p* = 0.012). **Conclusions:** Combined ultrasound-and-fluoroscopy guidance was associated with a significantly lower reintervention rate than ultrasound guidance alone, with consistent direction of effect across unadjusted and adjusted analyses. These findings support fluoroscopy as an adjunct modality for this technically demanding indication.

## 1. Introduction

Pleural effusion is among the most common thoracic conditions encountered in clinical practice, with an estimated annual incidence exceeding one million cases in the United States alone [1]. A clinically important subset—loculated pleural effusion—is characterized by fibrinous septations that divide the pleural space into compartmentalized collections, limiting spontaneous drainage and reducing the efficacy of simple thoracentesis. Loculation occurs in up to 30–40% of parapneumonic effusions and empyemas and is associated with higher rates of treatment failure, prolonged hospitalization, and the need for escalated intervention [1]. Percutaneous catheter drainage (PCD) under image guidance has become the standard first-line drainage strategy for loculated pleural effusion requiring intervention, offering an effective and minimally invasive alternative to surgical decortication [2,3,4].

In clinical practice, PCD for pleural effusion is performed under several image guidance approaches—ultrasound alone (US alone), fluoroscopy alone, or combined ultrasound and fluoroscopy (US + Fluoroscopy)—and the choice is not standardized, varying with operator preference and experience, patient condition, and the procedural setting (e.g., a dedicated interventional radiology suite versus a bedside procedure). Ultrasound guidance alone allows real-time needle visualization and is readily available at the bedside [5], whereas the addition of fluoroscopy provides real-time catheter advancement tracking and positional confirmation within the target locule. Although fluoroscopy introduces procedural complexity and radiation exposure, it may improve catheter placement accuracy—a particularly relevant advantage in loculated effusions where misplacement into an adjacent compartment can result in treatment failure.

Prior comparative studies of guidance modality in PCD have predominantly focused on unloculated or mixed pleural effusions, in which successful drainage is less dependent on precise catheter positioning [6,7,8]. In that context, the benefit of fluoroscopy over ultrasound alone was inconsistent across available evidence. Whether this finding extends to loculated pleural effusion—a technically more demanding subgroup—remains unclear, and no adequately adjusted head-to-head comparison in this specific population has been reported [9]. This gap in the evidence base represents the primary motivation for the present study.

Recognizing that guidance modality functions as a technical determinant of catheter positioning accuracy, and that loculation pattern and pleural fluid echogenicity may independently influence both drainage success and the relative benefit of fluoroscopic confirmation, we conducted the present study. The purpose of this study was to compare reintervention rates between ultrasound-guided and combined ultrasound-and-fluoroscopy-guided PCD in consecutive patients with loculated pleural effusion, after adjustment for key clinical and procedural confounders. We hypothesized that fluoroscopic guidance, by enabling real-time catheter positioning confirmation [10], would be associated with a lower reintervention rate compared with ultrasound guidance alone. Secondary aims were to characterize reintervention type, time to first reintervention, and procedural complication profiles in each group.

## 2. Materials and Methods

### 2.1. Study Design and Setting

This retrospective cohort study was conducted at a single tertiary-care academic medical center located in Changwon, Republic of Korea. All consecutive adult patients who underwent percutaneous catheter drainage (PCD) for loculated pleural effusion between January 2024 and December 2025 were screened for inclusion. The study was reported in accordance with the Strengthening the Reporting of Observational Studies in Epidemiology (STROBE) guidelines (Supplementary Materials). This study was approved by the Institutional Review Board of Samsung Changwon Hospital (approval no. SCMC IRB 2026-05-004, approved 18 May 2026). Informed consent was waived in accordance with the institutional policy for retrospective review of de-identified clinical data. The study was conducted in conformity with the principles of the Declaration of Helsinki.

### 2.2. Participants

**Figure 1:**
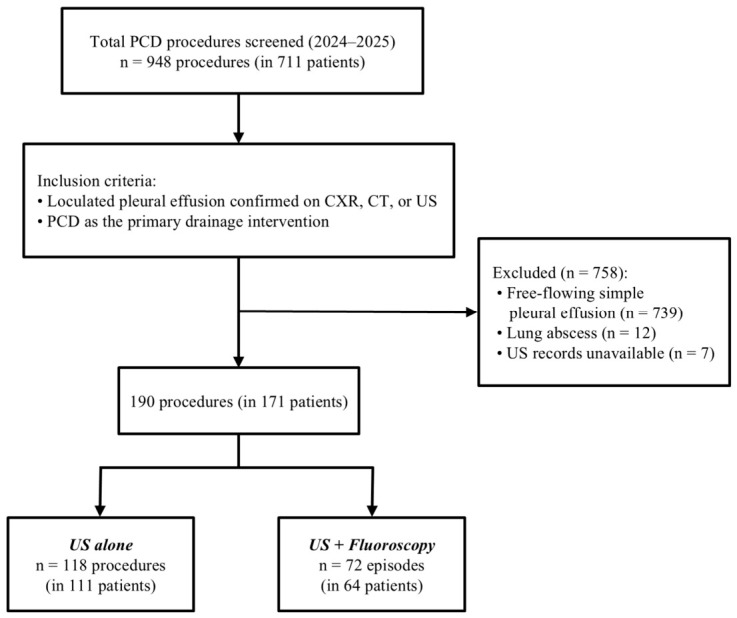
Patient selection flowchart. A total of 948 percutaneous catheter drainage (PCD) procedures in 711 patients were screened. After applying inclusion and exclusion criteria, 190 independent PCD procedures in 171 patients were enrolled: 118 in the ultrasound alone (US alone) group and 72 in the combined ultrasound-and-fluoroscopy (US + Fluoroscopy) group. An independent procedure was defined as PCD performed as the primary intervention for a loculated pleural effusion episode; a subsequent PCD was counted as a new independent procedure only if performed after clinical and radiological resolution of the preceding episode. CXR, chest radiograph; CT, computed tomography; US, ultrasound.

Inclusion and Exclusion: Patients were eligible if they (1) had a radiologically confirmed loculated pleural effusion on chest radiograph, computed tomography, or ultrasound, and (2) underwent PCD as the primary drainage intervention. Procedures were excluded if the effusion was free-flowing simple pleural effusion, if real-time ultrasound records were unavailable for review, or if drainage was performed for lung abscess. Reintervention catheters placed during the same clinical episode were not counted as independent procedures; however, a subsequent PCD performed after clinical and radiological recovery from a prior episode was considered an independent procedure and included. All eligible procedures were identified through the institutional procedural database. Patient selection is illustrated in Figure 1.

Group Assignment: Guidance modality was determined by the performing operator rather than by randomization. Two operators (Operator A and Operator B, with 6 and 15 years of interventional experience, respectively) performed all PCD procedures under ultrasound guidance alone (US alone group; *n* = 118 procedures). A third operator (Operator C, 3 years of interventional experience) performed all procedures under combined ultrasound and fluoroscopy guidance (US + Fluoroscopy group; *n* = 72 procedures). This operator-based assignment reflects established institutional workflow and is acknowledged as a potential source of confounding; its influence was addressed through multivariable adjustment and propensity score matching (see Section 2.5). Of the 171 patients, four contributed one procedure to each group during separate clinical episodes; accordingly, the procedure, rather than the patient, served as the unit of analysis, yielding 190 independent procedures in the final cohort.

### 2.3. PCD Procedure

US Alone Group: Procedures were performed in the interventional radiology suite with the patient positioned in the decubitus or sitting position on a stretcher. The target pleural locule was identified under real-time B-mode and/or color Doppler ultrasonography. Under continuous ultrasound guidance, the pleural space was accessed using an 18-gauge Medicut or Chiba needle via the Seldinger technique. A guidewire was advanced through the needle, followed by sequential tract dilation, and a pigtail catheter was inserted over the wire into the target locule. Post-procedure catheter position was confirmed by clinical assessment and portable chest radiograph.

**Figure 2:**
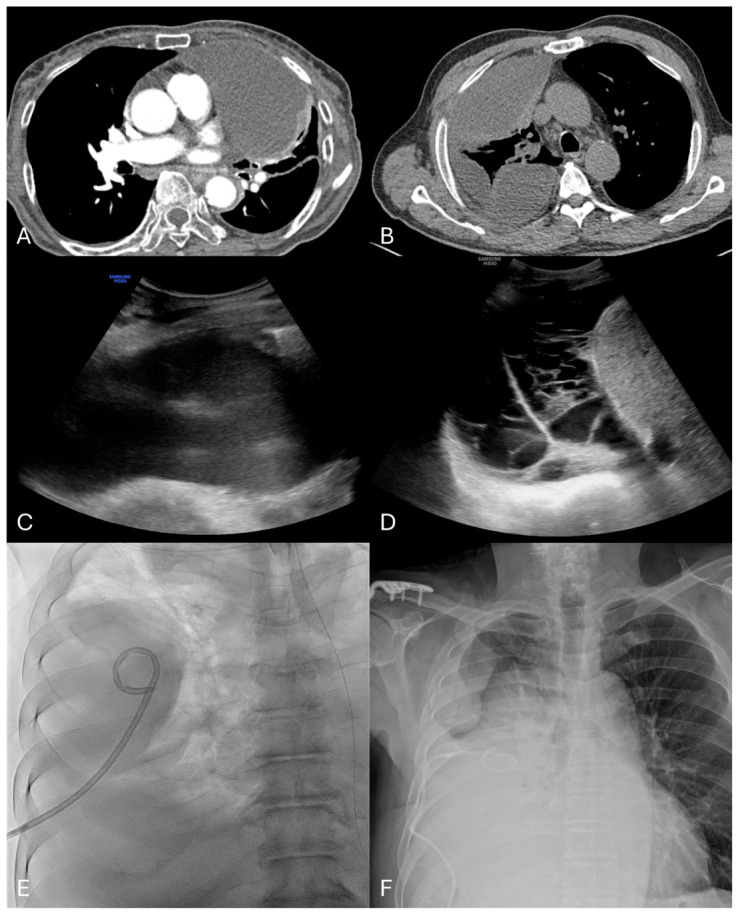
Representative imaging of study inclusion criteria and procedural guidance. (**A**) Computed tomography (CT) demonstrating a unilocular left-sided pleural effusion with a single well-defined locule. (**B**) CT demonstrating a multilocular right-sided pleural effusion. (**C**) Ultrasound (US) image showing an anechoic loculated pleural effusion. (**D**) Ultrasound image showing an echogenic pleural effusion with internal debris and septations. (**E**) Fluoroscopic image obtained during a US + Fluoroscopy-guided percutaneous catheter drainage procedure, confirming real-time pigtail catheter placement within the target pleural locule. (**F**) Post-procedure chest radiograph demonstrating satisfactory catheter position within the pleural space. All images are from representative study patients.

US + Fluoroscopy Group: Procedures were performed on the fluoroscopy table with the patient in the supine or decubitus position. Initial pleural access was obtained under real-time ultrasound guidance using the Seldinger technique, identical to the US alone group. Following guidewire placement, all subsequent steps—tract dilation and pigtail catheter advancement and positioning within the target locule—were performed under real-time fluoroscopic guidance using a C-arm unit. Fluoroscopy time was not formally recorded; however, based on operator records, exposure was typically less than 10 s per side per procedure. Post-procedure catheter position was confirmed by fluoroscopic image and chest radiograph (Figure 2).

Catheter and Effusion Characteristics: Pigtail drainage catheters from three manufacturers were used across both groups: SKATER™ drainage catheter (Argon Medical Devices, Athens, TX, USA), Dawson–Mueller multipurpose drainage catheter (Cook Medical, Bloomington, IN, USA), and multipurpose drainage catheter II (Sungwon Medical, Cheongju, Republic of Korea). Catheter size (8–8.5 Fr or 10–10.2 Fr) was selected at the operator’s discretion based on effusion characteristics. Loculation pattern (unilocular versus multilocular) and pleural fluid echogenicity (anechoic versus echogenic/debris) were assessed by the performing operator at the time of the procedure. Interobserver agreement for these assessments was evaluated in a subset of cases reviewed independently by one interventional radiologist and one chest radiologist, and was almost perfect for both echogenicity (Cohen’s κ = 0.832) and loculation pattern (Cohen’s κ = 0.820) [11].

### 2.4. Outcome Measures

Primary Outcome: The primary outcome was any reintervention, defined as a requirement for catheter repositioning or replacement during the same clinical episode due to catheter malposition, catheter displacement, drainage failure, or development of a new loculation [12,13].

Initial Technical Success: Initial technical success was defined as immediate aspiration of pleural fluid upon catheter placement, confirmed before procedure completion.

Secondary Outcomes: Secondary outcomes included: (1) reintervention type (malposition, displacement, drainage failure, or new loculation), categorized according to the clinical indication for the procedure; (2) time to first reintervention, measured in days from the first PCD procedure of the episode; and (3) procedural complication rate, including pneumothorax and major complications (defined as pneumothorax, hemothorax, or visceral perforation requiring intervention). No intrapleural fibrinolytic therapy was administered in either group during the study period.

### 2.5. Statistical Analysis

Categorical variables were summarized as frequencies and percentages and compared between groups using Fisher’s exact test (for 2 × 2 tables) or the chi-square test. Continuous variables were summarized as medians with interquartile ranges (IQR) and compared using the Mann–Whitney U test. The relative risk (RR) for the primary outcome with its 95% confidence interval (CI) was estimated using the log-normal approximation. Missing values for laboratory variables were infrequent (detailed in Table 1) and handled by complete-case analysis; no laboratory variables were included in the primary regression models.

Multivariable Logistic Regression: To adjust for potential confounders, we fitted a multivariable binary logistic regression model with reintervention as the dependent variable. Independent variables were guidance group (US alone as reference), age, sex, loculation pattern, pleural fluid echogenicity, catheter French size, and clinical etiology (categorized as parapneumonic effusion [reference], empyema, malignant effusion, or other [tuberculous, postoperative, or miscellaneous]). Results are reported as adjusted odds ratios (aOR) with 95% CIs.

Propensity Score Matching: To further account for baseline differences between groups, we performed 1:1 nearest-neighbor propensity score matching (PSM) without replacement, using a caliper width of 0.2 standard deviations of the logit-transformed propensity score [14]. Propensity scores were estimated using logistic regression with the following covariates: age, sex, loculation pattern, echogenicity, and clinical etiology. Clinical etiology was grouped into four categories (parapneumonic, empyema, malignant, and other [postoperative, tuberculous, or miscellaneous]) in both the propensity score and outcome regression models to avoid sparse-cell instability. Catheter French size was excluded from the propensity model as it reflected operator judgment after effusion assessment rather than an independent patient characteristic. Post-match covariate balance was assessed using standardized mean differences (SMD), with SMD < 0.1 considered indicative of adequate balance. Reintervention rates in the matched cohort were compared using McNemar’s test for matched pairs.

Time-to-Event Analysis: Kaplan–Meier curves for time to first reintervention were constructed for each group. Patients without reintervention were censored at the maximum observed follow-up duration in the cohort (38 days). Curves were compared using the log-rank test. A multivariable Cox proportional hazards model was fitted using the same covariate set as the logistic regression model; results are reported as adjusted hazard ratios (aHR) with 95% CIs. Model discrimination was assessed using the concordance index.

All statistical analyses were performed using R (version 4.6.0; R Foundation for Statistical Computing, Vienna, Austria) with the survival (3.8-6), MatchIt (4.7.2), and epitools (0.5-10.1) packages. Kaplan–Meier curves were visualized using the survminer (0.5.2) package. All tests were two-sided, with a significance threshold of α = 0.05.

## 3. Results

### 3.1. Study Population

A total of 948 PCD procedures performed in 711 patients between January 2024 and December 2025 were screened for inclusion. After excluding 758 procedures (free-flowing simple pleural effusion, *n* = 739; lung abscess, *n* = 12; ultrasound records unavailable, *n* = 7) and applying the episode definition described in Section 2.2, 190 independent PCD procedures in 171 patients (four of whom contributed procedures to both groups during separate episodes) were included in the final analytic cohort: 118 procedures in the US alone group and 72 procedures in the US + Fluoroscopy group (Figure 1). Baseline characteristics are presented in Table 1. Median age was 71.0 years (IQR 62.2–76.0) in the US alone group and 75.0 years (IQR 64.0–80.0) in the US + Fluoroscopy group (*p* = 0.026); the US + Fluoroscopy group was significantly older. The two groups were otherwise comparable in sex distribution, etiology, loculation pattern, and pleural fluid echogenicity (Table 1). In the US alone group, all 118 procedures were performed using 8–8.5 Fr catheters; in the US + Fluoroscopy group, 8–8.5 Fr catheters were used in 55 procedures (76.4%) and 10–10.2 Fr catheters in 17 procedures (23.6%), a significant difference in catheter size distribution (*p* < 0.001) (Table 1). Laboratory values, including pleural fluid parameters, are detailed in Table 1.

### 3.2. Primary Outcome—Reintervention Rate

Initial technical success was achieved in all 190 procedures (100%). Reintervention occurred in 42 of 118 procedures (35.6%) in the US alone group and in 13 of 72 procedures (18.1%) in the US + Fluoroscopy group (*p* = 0.013; RR 0.51, 95% CI 0.29–0.88) (Table 2). The distribution of reintervention types differed between groups: catheter malposition and displacement accounted for the majority of reinterventions in the US alone group, whereas drainage failure and new loculation comprised a greater proportion in the US + Fluoroscopy group (Table 2).

### 3.3. Time to Reintervention

**Figure 3:**
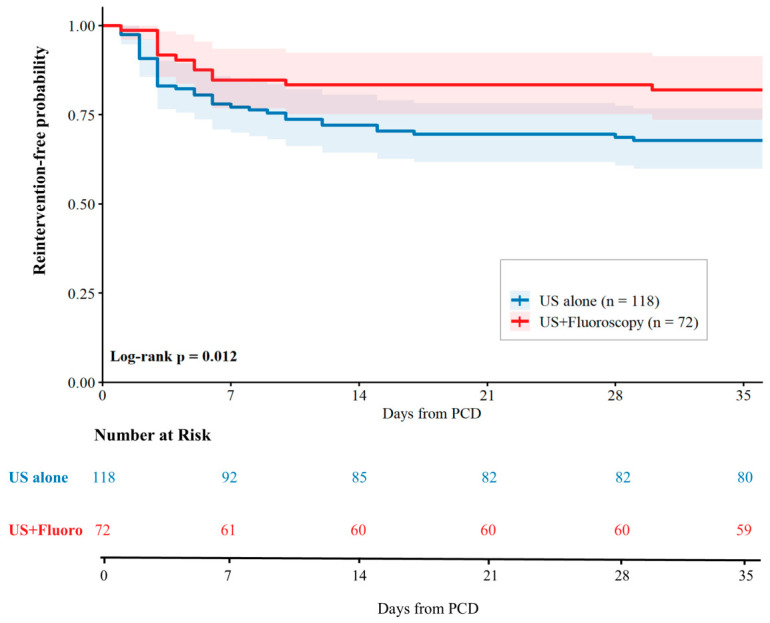
Kaplan–Meier curves for time to first reintervention. The US alone group (blue) and US + Fluoroscopy group (red) are shown with 95% confidence intervals (shaded bands). Numbers at risk are displayed below the *x*-axis at 0, 7, 14, 21, 28, and 35 days. Unadjusted log-rank *p* = 0.012. Curves generated using the survminer package (R version 4.6.0). PCD, percutaneous catheter drainage; US, ultrasound.

Among patients who underwent reintervention, the median time to first reintervention was 3.0 days (IQR 2.0–9.5) in the US alone group and 4.0 days (IQR 3.0–6.0) in the US + Fluoroscopy group; this difference was not statistically significant (Mann–Whitney U, *p* = 0.886). Kaplan–Meier reintervention-free survival curves are presented in Figure 3; the unadjusted log-rank test demonstrated a significant difference in time-to-reintervention between groups (*p* = 0.012).

### 3.4. Adjusted Analyses

Multivariable Logistic Regression: After adjustment for age, sex, loculation pattern, echogenicity, catheter French size, and clinical etiology, US + Fluoroscopy guidance was associated with significantly lower odds of reintervention compared with US alone (aOR 0.42, 95% CI 0.17–0.95; *p* = 0.043) (Table 3A).

Propensity Score Matching: Propensity score matching yielded 60 matched pairs. Post-match standardized mean differences were below 0.10 for all covariates except the malignant etiology subgroup (SMD = 0.107), indicating acceptable overall covariate balance. Within the matched cohort, reintervention occurred in 21 of 60 procedures (35.0%) in the US alone group and in 10 of 60 procedures (16.7%) in the US + Fluoroscopy group (risk difference 18.3%, 95% CI 2.7–34.0%; McNemar’s *p* = 0.046) (Table 3B).

Cox Proportional Hazards Model: In the multivariable Cox proportional hazards model, US + Fluoroscopy guidance was associated with a lower hazard of reintervention compared with US alone, although the 95% confidence interval marginally crossed unity (aHR 0.50, 95% CI 0.25–1.02; *p* = 0.056). Model discrimination was moderate (concordance index = 0.719, indicating moderate discriminative ability) (Table 3C).

Procedural complication data are summarized in Table 4. No minor complications were recorded in either group. The sole major complication was pneumothorax requiring chest tube insertion, occurring in 1 of 118 procedures (0.8%) in the US alone group and in 1 of 72 procedures (1.4%) in the US + Fluoroscopy group (*p* = 1.000 by Fisher’s exact test).

## 4. Discussion

In this retrospective cohort study of 190 independent percutaneous catheter drainage procedures for loculated pleural effusion, combined ultrasound and fluoroscopy guidance was associated with a significantly lower reintervention rate compared with ultrasound guidance alone (18.1% versus 35.6%; relative risk 0.51, 95% CI 0.29–0.88). This association persisted after multivariable adjustment (adjusted odds ratio 0.42, 95% CI 0.17–0.95) and propensity score matching (35.0% versus 16.7%; McNemar’s *p* = 0.046), and was directionally consistent in the time-to-event analysis, although the Cox model 95% confidence interval marginally crossed unity (adjusted hazard ratio 0.50, 95% CI 0.25–1.02). Taken together, the consistent direction of effect across all analytical approaches suggests that fluoroscopic guidance adds clinically meaningful value to ultrasound-guided PCD in patients with loculated pleural effusion.

The observed benefit of fluoroscopy is consistent with its mechanical role in the procedure: unlike free-flowing effusion—where catheter placement anywhere within the pleural space typically achieves drainage—loculated effusion requires the catheter tip to be accurately positioned within a specific locule. When ultrasound guidance alone is used, real-time catheter visualization is limited after the needle enters the pleural space, and final catheter position is verified retrospectively by chest radiograph. In the current cohort, catheter malposition and displacement accounted for the predominant reintervention types in the US alone group, and the median time to first reintervention was three days—consistent with early positional failure rather than delayed drainage failure from disease progression [12].

Prior studies evaluating guidance modality for PCD have largely focused on unloculated or mixed pleural effusions, where the technical demands of catheter positioning are lower [6,7,15]. To our knowledge, adequately adjusted comparisons specifically restricted to loculated pleural effusion—the subset in which positional accuracy is most consequential—have not been previously reported [9]. The present study addresses this gap in a consecutive institutional cohort with systematic collection of procedural variables.

A key consideration in interpreting these results is the non-random allocation of guidance modality, which was determined by the performing operator. The two US alone operators had 6 and 15 years of interventional experience, respectively, whereas the US + Fluoroscopy operator had 3 years of experience at the time of the study. The direction of this experience imbalance favors the US alone group, in that the more experienced operators used the simpler guidance modality. Despite this disadvantage, the US + Fluoroscopy group demonstrated a lower reintervention rate that remained statistically significant after propensity score matching and multivariable adjustment for all available baseline characteristics. This pattern argues against operator skill as the primary explanation for the observed difference, and supports fluoroscopy as a procedurally important adjunct independent of operator seniority. A randomized controlled trial stratified by operator experience would be needed to fully disentangle these contributions, but the consistency of the association across multiple analytical methods strengthens its plausibility.

Notably, the US + Fluoroscopy group was significantly older than the US alone group at baseline (median 75 versus 71 years; *p* = 0.026). Although a 4-year difference in median age is clinically modest, older age is generally associated with greater procedural complexity and a higher likelihood of reintervention. The fact that the US + Fluoroscopy group demonstrated a lower reintervention rate despite this unfavorable age profile argues against age as a confounding explanation for the observed benefit, and reinforces the robustness of the guidance modality effect. Age was included as a covariate in all multivariable models.

The clinical implications of these findings are most relevant for complex loculated effusions, particularly multilocular effusions in which drainage of the target locule requires accurate catheter positioning that may be difficult to confirm under ultrasound alone. Malignant pleural effusions, which exhibit higher intrinsic reintervention rates regardless of drainage technique due to ongoing fluid production and loculation progression [1], were similarly distributed between groups in the matched cohort. Although the current study enrolled loculated effusions of diverse etiologies (parapneumonic, empyema, malignant, and other causes), all share the fundamental technical challenge of septation navigation that renders catheter positioning accuracy the key determinant of drainage success, regardless of underlying cause. The current study was not powered to formally test for interaction between guidance modality and loculation pattern or etiology; however, the overall consistency of results across analytical methods suggests that the guidance effect is not driven by a single subgroup. For institutions considering protocol development, fluoroscopy-guided PCD may be particularly appropriate for patients with multilocular effusions or echogenic/debris-containing fluid, both of which are associated with increased technical difficulty and reintervention risk, although subgroup-specific benefits remain to be confirmed in larger prospective studies [16,17,18].

However, using fluoroscopy also comes with practical drawbacks that need to be considered. Compared with ultrasound-alone PCD, fluoroscopy-guided procedures require a fluoroscopy suite rather than a bedside or portable setting, limiting availability in critically ill patients who cannot be safely transported. Procedure time is modestly prolonged by table setup and C-arm positioning. Radiation exposure, while low for individual procedures (typically less than 10 s of fluoroscopy time in the present series), may be cumulative for patients requiring multiple interventions [19]. Equipment and facility costs are slightly higher. Although the addition of fluoroscopy entails a modestly higher cost, its association with a lower reintervention rate may translate into meaningful clinical and economic benefits, including reduced discomfort and morbidity from repeat procedures and potentially lower overall patient care costs.

Several limitations warrant consideration. First, the retrospective, single-center design introduces selection bias and limits generalizability. Patients were not randomly assigned to guidance modality, and unmeasured confounders may have influenced the results. Guidance modality was closely associated with operator practice: all ultrasound–fluoroscopy procedures were performed by a single operator, whereas ultrasound-only procedures were performed by two other operators. The independent effect of fluoroscopic guidance could not be fully separated from operator-related factors, including access site selection, guidewire manipulation, and final catheter positioning. Propensity score matching and multivariable regression reduced measurable imbalances, but residual confounding, including operator-related bias, cannot be excluded.

Second, the ultrasound–fluoroscopy group was relatively small, which may have limited statistical power in the time-to-event analysis. The Cox regression results should therefore be interpreted in conjunction with the logistic regression and propensity score–matched analyses. Third, radiation exposure was not prospectively measured; fluoroscopy time was typically less than 10 s per procedure based on operator records, consistent with a low radiation burden. Fourth, censoring for time-to-event analysis was performed at the maximum observed follow-up period rather than individually documented clinical follow-up dates, which may have introduced bias in the estimation of time to reintervention.

## 5. Conclusions

In 190 independent PCD procedures for loculated pleural effusion, combined ultrasound and fluoroscopy guidance was associated with a significantly lower reintervention rate compared with ultrasound guidance alone, an association that was robust across unadjusted, multivariable-adjusted, and propensity score-matched analyses. These results support consideration of fluoroscopy as an adjunct guidance modality for this technically demanding indication, particularly in patients with multilocular or echogenic effusions in whom accurate catheter positioning is most critical. Prospective studies with randomized guidance allocation are warranted to confirm these findings and to evaluate potential subgroup effects.

## Figures and Tables

**Table 1:** Baseline Characteristics of Study Groups.

Variable	US Alone (*n* = 118)	US + Fluoroscopy (*n* = 72)	*p*-Value
Age, years	71.0 (62.2–76.0)	75.0 (64.0–80.0)	0.026
Sex, male	85 (72.0%)	55 (76.4%)	0.611
Clinical diagnosis, *p* = 0.795			
Parapneumonic	28 (23.7%)	21 (29.2%)	
Empyema	18 (15.3%)	11 (15.3%)	
Malignant	45 (38.1%)	23 (31.9%)	
Other	27 (22.9%)	17 (23.6%)	
Loculation, multilocular	61 (51.7%)	37 (51.4%)	1.000
Echogenicity, echogenic/debris	63 (53.4%)	33 (45.8%)	0.370
Catheter size, *p* = <0.001			
8–8.5 Fr	118 (100.0%)	55 (76.4%)	
10–10.2 Fr	0 (0.0%)	17 (23.6%)	
WBC, ×10^3^/μL	9.8 (6.8–15.2)	9.0 (6.7–13.7)	0.760
CRP, mg/L [2/0 missing]	76.6 (33.2–174.7)	80.1 (33.5–154.0)	0.931
Serum albumin, g/dL [1/0 missing]	3.1 (2.8–3.5)	3.2 (2.8–3.6)	0.479
Pleural pH [11/8 missing]	7.4 (7.2–7.5)	7.4 (7.2–7.5)	0.995
Pleural protein, g/dL [4/3 missing]	3.9 (3.1–4.7)	3.9 (2.8–4.5)	0.209
Pleural glucose, mg/dL [16/6 missing]	114.0 (79.2–136.0)	119.0 (72.0–140.5)	0.723
Pleural LDH, IU/L [21/10 missing]	358.0 (191.0–717.0)	271.0 (132.0–704.2)	0.077

Data presented as median (interquartile range) for continuous variables or *n* (%) for categorical variables. *p*-values: Mann–Whitney U test for continuous variables; Fisher’s exact test for categorical variables. Missing values are noted in brackets where applicable. WBC = White blood cell count; CRP = C-reactive protein; LDH = lactate dehydrogenase.

**Table 2:** Reintervention Rates and Types by Guidance Group.

Variable	US Alone (*n* = 118)	US + Fluoroscopy (*n* = 72)	*p*-Value/RR
Any reintervention	42 (35.6%)	13 (18.1%)	0.013
Catheter malposition	4 (3.4%)	0 (0.0%)	
Catheter displacement	3 (2.5%)	2 (2.8%)	
Drainage failure	24 (20.3%)	7 (9.7%)	
New loculation	12 (10.2%)	4 (5.6%)	
Relative risk (95% CI)			0.507 (0.293–0.878)
Time to reintervention, days *	3.0 (2.0–9.5)	4.0 (3.0–6.0)	0.886

*p*-value for any reintervention: Fisher’s exact test. One procedure in the US alone group was coded as both drainage failure and new loculation and is counted in both type categories. * Among procedures with reintervention only; presented as median (IQR). RR = relative risk; CI = confidence interval.

**Table 3:** (**A**) Multivariable Logistic Regression: Adjusted Odds Ratios for Reintervention. (**B**) Propensity Score Matching: Covariate Balance and Matched Outcome. (**C**) Multivariable Cox Proportional Hazards Model: Adjusted Hazard Ratios for Time to Reintervention.

(**A**)				
**Variable**	**aOR**	**95% CI Lower**	**95% CI Upper**	***p*** **-Value**
US + Fluoroscopy vs. US alone	0.42	0.17	0.95	0.043
Age (per year)	1.03	1.00	1.07	0.047
Sex, male	1.64	0.71	3.99	0.257
Loculation, multilocular	2.57	1.26	5.41	0.011
Echogenicity, echogenic/debris	2.18	1.03	4.71	0.043
Catheter size, 10–10.2 Fr vs. 8–8.5 Fr	0.37	0.07	1.65	0.217
Etiology: Parapneumonic (ref)	1.00			ref
Etiology: Empyema	1.90	0.62	5.91	0.261
Etiology: Malignant	1.79	0.71	4.70	0.225
Etiology: Other (postop/Tbc/misc)	0.77	0.27	2.17	0.623
(**B**)				
**Part A. Covariate Balance**				
**Covariate**	**SMD Before Matching**		**SMD After Matching**	
Propensity score	0.359		0.001	
Age	0.312		0.103	
Sex, male	0.103		0.157	
Loculation, multilocular	0.006		0.033	
Echogenicity, echogenic/debris	0.152		0.033	
Etiology: Parapneumonic	0.120		0.037	
Etiology: Empyema	0.001		0.046	
Etiology: Malignant	0.133		0.107	
Etiology: Other	0.017		0.039	
**Part B. Matched Cohort Outcome**				
**Variable**	**Value**			
Matched pairs	60			
Reintervention, US alone	21/60 (35.0%)			
Reintervention, US + Fluoroscopy	10/60 (16.7%)			
Risk difference (US alone − US+F)	18.3% (95% CI 2.7–34.0%)			
McNemar *p*-value	0.046			
(**C**)				
**Variable**	**aHR**	**95% CI Lower**	**95% CI Upper**	***p*** **-Value**
US + Fluoroscopy vs. US alone	0.50	0.25	1.02	0.056
Age (per year)	1.03	1.00	1.06	0.027
Sex, male	1.39	0.70	2.74	0.347
Loculation, multilocular	2.43	1.33	4.41	0.004
Echogenicity, echogenic/debris	1.93	1.02	3.62	0.042
Catheter size, 10–10.2 Fr vs. 8–8.5 Fr	0.47	0.12	1.81	0.270
Etiology: Parapneumonic (ref)	1.00			ref
Etiology: Empyema	1.46	0.64	3.35	0.373
Etiology: Malignant	1.61	0.75	3.47	0.225
Etiology: Other (postop/Tbc/misc)	0.80	0.34	1.89	0.614

(**A**) Reference categories: guidance group = US alone; sex = female; catheter size = 8–8.5 Fr; etiology = parapneumonic effusion. aOR = adjusted odds ratio; CI = confidence interval. (**B**) SMD = standardized mean difference. SMD < 0.10 indicates adequate covariate balance. Matching: 1:1 nearest-neighbor, caliper 0.2 SD of logit (propensity score), without replacement. Reintervention compared by McNemar’s test for matched pairs. Risk difference CI calculated by the Nam method for paired proportions. (**C**) Reference categories as in Table 3A. Patients without reintervention were censored at 38 days (maximum observed follow-up). Concordance index = 0.719. aHR = adjusted hazard ratio; CI = confidence interval.

**Table 4:** Procedural Safety Outcomes.

Complication	US Alone (*n* = 118)	US + Fluoroscopy (*n* = 72)	*p*-Value
Minor complications	0 (0.0%)	0 (0.0%)	N/A
Major complications			
Pneumothorax requiring chest tube insertion	1 (0.8%)	1 (1.4%)	1.000

Complications classified in accordance with the Society of Interventional Radiology (SIR) classification system. Minor: No therapy or nominal therapy required. Major: Requires active intervention or hospitalization. Both pneumothorax events required chest tube insertion. *p*-value by Fisher’s exact test; N/A = no events in either group.

## Data Availability

The data that support the findings of this study are available from the corresponding author upon reasonable request. Data are not publicly available owing to patient privacy considerations.

## Supplementary Materials

The following supporting information can be downloaded at: https://www.mdpi.com/article/10.3390/diagnostics16132089/s1, STROBE Checklist—Cohort Study.

## References

[1] Light R.W. (2002). Pleural effusion. N. Engl. J. Med..

[2] Dariushnia S.R., Mitchell J.W., Chaudry G., Hogan M.J. (2020). Society of Interventional Radiology Quality Improvement Standards for Image-Guided Percutaneous Drainage and Aspiration of Abscesses and Fluid Collections. J. Vasc. Interv. Radiol..

[3] Bedawi E.O., Ricciardi S., Hassan M., Gooseman M.R., Asciak R., Castro-Anon O., Armbruster K., Bonifazi M., Poole S., Harris E.K. (2023). ERS/ESTS statement on the management of pleural infection in adults. Eur. Respir. J..

[4] Roberts M.E., Rahman N.M., Maskell N.A., Bibby A.C., Blyth K.G., Corcoran J.P., Edey A., Evison M., de Fonseka D., Hallifax R. (2023). British Thoracic Society Guideline for pleural disease. Thorax.

[5] Chang W.H., Lee C., Kuo L.K., Yu K.P. (2026). Real-time ultrasound-guided versus ultrasound-assisted pigtail catheter insertion for pleural effusion drainage in the intensive care unit: A retrospective cohort study. J. Intensive Care Med..

[6] Keeling A.N., Leong S., Logan P.M., Lee M.J. (2008). Empyema and effusion: Outcome of image-guided small-bore catheter drainage. Cardiovasc. Interv. Radiol..

[7] Liu Y.H., Lin Y.C., Liang S.J., Tu C.Y., Chen C.H., Chen H.J., Chen W., Shih C.-M., Hsu W.-H. (2010). Ultrasound-guided pigtail catheters for drainage of various pleural diseases. Am. J. Emerg. Med..

[8] Kiranantawat N., Sungsiri J., Geater S.L. (2014). Outcome of ultrasound-guided small-bore catheter drainage in exudative pleural effusions. J. Med. Assoc. Thai..

[9] Rafiq S., Dar M.A., Nazir I., Shaffi F., Shaheen F., Kuchay I.A. (2020). Image-guided catheter drainage in loculated pleural space collections, effectiveness, and complications. Lung India.

[10] Havelock T., Teoh R., Laws D., Gleeson F. (2010). Pleural procedures and thoracic ultrasound: British Thoracic Society Pleural Disease Guideline 2010. Thorax.

[11] Landis J.R., Koch G.G. (1977). The measurement of observer agreement for categorical data. Biometrics.

[12] Alomar Z., Tawfek Z., Alomar Y., Mahmood I., Alomar A., El-Menyar A., Rizoli S., Al-Thani H. (2025). Failure rate and complications of small-bore, wire-guided chest drains in adult patients presenting with traumatic and nontraumatic pleural diseases: A systematic review. Qatar Med. J..

[13] Cafarotti S., Dall’Armi V., Cusumano G., Margaritora S., Meacci E., Lococo F., Vita M., Porziella V., Bonassi S., Cesario A. (2011). Small-bore wire-guided chest drains: Safety, tolerability, and effectiveness in pneumothorax, malignant effusions, and pleural empyema. J. Thorac. Cardiovasc. Surg..

[14] Austin P.C. (2011). An Introduction to Propensity Score Methods for Reducing the Effects of Confounding in Observational Studies. Multivar. Behav. Res..

[15] Akhan O., Ozkan O., Akinci D., Hassan A., Ozmen M. (2007). Image-guided catheter drainage of infected pleural effusions. Diagn. Interv. Radiol..

[16] Jayakrishnan B., Kashoob M., Al-Sukaiti R., Al-Mubaihsi S., Kakaria A., Al-Ghafri A., Al-Lawati Y. (2021). Percutaneous Ultrasound-guided Pigtail Catheter for Pleural Effusions: Efficacy and Safety. Oman Med. J..

[17] De Gregorio M.A., Ruiz C., Alfonso E.R., Fernandez J.A., Medrano J., Arino I. (1999). Transcatheter intracavitary fibrinolysis of loculated pleural effusions: Experience in 102 patients. Cardiovasc. Interv. Radiol..

[18] Park C.S., Chung W.M., Lim M.K., Cho C.H., Suh C.H., Chung W.K. (1996). Transcatheter instillation of urokinase into loculated pleural effusion: Analysis of treatment effect. AJR Am. J. Roentgenol..

[19] Cantin L., Chartrand-Lefebvre C., Lepanto L., Gianfelice D., Rabbat A., Aubin B., Perreault P., Dery R., Lafortune M. (2005). Chest tube drainage under radiological guidance for pleural effusion and pneumothorax in a tertiary care university teaching hospital: Review of 51 cases. Can. Respir. J..

