# Supplementary material for: Ultrasound–Fluoroscopy Versus Ultrasound Guidance for Catheter Drainage of Loculated Pleural Effusions: A Retrospective Study
Source: Diagnostics (Basel). 2026 Jul 3;16(13):2089. doi: 10.3390/diagnostics16132089 (PMC13360030; doi:10.3390/diagnostics16132089)
Supplement: Supplementary file 1 [file diagnostics-16-02089-s001.zip › diagnostics-4390402-supplementary.pdf]

STROBE Checklist — Cohort Study

Ultrasound-Alone Versus Combined Ultrasound-and-Fluoroscopy-Guided PCD for Loculated Pleural Effusion: A Comparative Study of Reintervention Rates

Section/Topic	Item	Recommendation	Reported?	Location in Manuscript
**Title and Abstract**	1	(a) Indicate the study's design with a commonly used term in the title or abstract<br>(b) Provide in the abstract an informative and balanced summary of what was done and what was found	**Yes**	Title includes 'Comparative Study'; Abstract structured with Background/Methods/Results/Conclusions
**Background/rationale**	2	Explain the scientific background and rationale for the investigation being reported	**Yes**	Introduction paragraphs 1–2; cites Light 2002, Dariushnia 2020, Rafiq 2020
**Objectives**	3	State specific objectives, including any prespecified hypotheses	**Yes**	Introduction paragraph 3: reintervention rate comparison; hypothesis stated
**Study design**	4	Present key elements of study design early in the paper	**Yes**	Methods 2.1: 'retrospective cohort study'
**Setting**	5	Describe the setting, locations, and relevant dates, including periods of recruitment, exposure, follow-up, and data collection	**Yes**	Methods 2.1: single tertiary-care center; Jan 2024–Dec 2025
**Participants**	6	(a) Cohort study — give the eligibility criteria, and the sources and methods of selection of participants; describe methods of follow-up<br>(b) For matched studies, give matching criteria and number of exposed and unexposed	**Yes**	Methods 2.2: inclusion/exclusion criteria, operator-based assignment; n=118 and n=72; Figure 1 flowchart
**Variables**	7	Clearly define all outcomes, exposures, predictors, potential confounders, and effect modifiers; give diagnostic criteria, if applicable	**Yes**	Methods 2.3–2.4: catheter guidance modality (exposure), reintervention (primary outcome), covariates listed in 2.5
**Data sources/measurement**	8	For each variable of interest, give sources of data and details of methods of assessment; describe comparability of assessment methods if there is more than one group	**Yes**	Methods 2.3: US and fluoroscopy procedures described; 2.3: interobserver agreement kappa reported
**Bias**	9	Describe any efforts to address potential sources of bias	**Yes**	Methods 2.5: PSM and multivariable regression; Discussion P3: operator experience confounding discussed
**Study size**	10	Explain how the study size was arrived at	**Partial**	Results 3.1: consecutive eligible episodes reported; no formal sample size calculation (retrospective)
**Quantitative variables**	11	Explain how quantitative variables were handled in the analyses; if applicable, describe which groupings were chosen and why	**Yes**	Methods 2.5: continuous variables as medians/IQR; categorical groupings defined in 2.3–2.4
**Statistical methods (a)**	12a	Describe all statistical methods, including those used to control for confounding	**Yes**	Methods 2.5: Mann-Whitney, Fisher's exact, log-normal RR, logistic regression, PSM (MatchIt), Cox PH, R 4.6.0
**Statistical methods (b)**	12b	Describe any methods used to examine subgroups and interactions	**Yes**	Methods 2.5 / Discussion P4: subgroup effects acknowledged; formal interaction not tested (acknowledged in P5)
**Statistical methods (c)**	12c	Explain how missing data were addressed	**Yes**	Methods 2.5: complete-case analysis; laboratory variables excluded from primary regression models
**Statistical methods (d)**	12d	If applicable, explain how loss to follow-up was addressed	**Yes**	Methods 2.5 / Discussion P5: censoring at max follow-up (38 days); informative censoring limitation acknowledged
**Statistical methods**	12e	Describe any sensitivity analyses	**Partial**	No formal sensitivity analysis; borderline

**(e)**				Cox result interpreted contextually in Discussion P5
**Participants**	13	(a) Report numbers of individuals at each stage of study — numbers potentially eligible, examined for eligibility, confirmed eligible, included, completing follow-up, analysed<br>(b) Give reasons for non-participation at each stage<br>(c) Consider use of a flow diagram	**Yes**	Results 3.1: 948 screened → 758 excluded → 190 included; Figure 1 STROBE flowchart
**Descriptive data**	14	(a) Give characteristics of study participants and information on exposures and potential confounders<br>(b) Indicate number of participants with missing data for each variable of interest<br>(c) Summarise follow-up time	**Yes**	Results 3.1: Table 1 baseline characteristics; missing values noted in Table 1 footnote; follow-up censored at 38 days
**Outcome data**	15	Report numbers of outcome events or summary measures over time	**Yes**	Results 3.2: 42/118 vs 13/72; Table 2 reintervention types; Results 3.3: time to reintervention
**Main results**	16	(a) Give unadjusted estimates and, if applicable, confounder-adjusted estimates and their precision<br>(b) Report category boundaries when continuous variables were categorized<br>(c) If relevant, consider translating estimates of relative risk into absolute risk for a meaningful time period	**Yes**	Results 3.2: RR 0.51 (95% CI 0.29-0.88); Results 3.4: aOR 0.42, PSM McNemar p=0.046, aHR 0.50; Tables 3A/3B/3C
**Other analyses**	17	Report other analyses done — e.g., analyses of subgroups and interactions, and sensitivity analyses	**Partial**	Results 3.3: Kaplan-Meier + log-rank; Results 3.4: three adjusted analyses presented; no separate sensitivity analysis
**Key results**	18	Summarise key results with reference to study objectives	**Yes**	Discussion P1
**Limitations**	19	Discuss limitations of the study, taking into account sources of potential bias or imprecision; discuss both direction and magnitude of any potential bias	**Yes**	Discussion P5: retrospective design, small US+Fluoro group, unmeasured confounders, censoring
**Interpretation**	20	Give a cautious overall interpretation of results considering objectives, limitations, multiplicity of analyses, and results from similar studies	**Yes**	Discussion P2–P4: mechanism, prior literature, operator confounding, clinical implications
**Generalisability**	21	Discuss the generalisability (external validity) of the study results	**Yes**	Discussion P5: single-center, operator-based allocation; P6 Conclusion notes prospective studies needed
**Funding**	22	Give the source of funding and the role of the funders for the present study and, if applicable, for the original study on which the present article is based	**Yes**	Funding section: 'This research received no external funding.'

Reporting status: Yes = fully reported | Partial = partially addressed | No = not reported. STROBE = Strengthening the Reporting of Observational Studies in Epidemiology.
